# CT定量参数预测肺磨玻璃结节病理类型的价值

**DOI:** 10.3779/j.issn.1009-3419.2024.102.09

**Published:** 2024-02-20

**Authors:** Yiqiu SHI, Yuwen SHEN, Jie CHEN, Wanying YAN, Kefu LIU

**Affiliations:** ^1^215008 苏州，南京医科大学附属苏州医院，苏州市立医院放射科（石逸秋，沈雨雯，陈劼，刘可夫）; ^1^Department of Radiology, Suzhou Municipal Hospital, The Affiliated Suzhou Hospital of Nanjing Medical University, Suzhou 215008, China; ^2^100020 北京，推想医疗科技股份有限公司（闫婉莹）; ^2^Infervision Medical Technology Co., Ltd, Beijing 100020, China

**Keywords:** 肺肿瘤, 磨玻璃结节, 病理类型, 计算机断层扫描, Lung neoplasms, Ground glass nodules, Pathological type, Computed tomography

## Abstract

**背景与目的** 肺磨玻璃结节（ground glass nodules, GGNs）的病理类型对临床治疗方案的选择具有十分重要的意义，本研究旨在探讨主观计算机断层扫描（computed tomography, CT）影像学征象及人工智能定量参数在预测GGNs病理类型中的价值。**方法** 回顾性分析389例病理明确诊断的GGNs，其中，前驱腺体病变[包括非典型瘤样增生（atypical adenomatous hyperplasia, AAH）、原位腺癌（adenocarcinoma in situ, AIS）]138例，微浸润腺癌（microinvasive adenocarcinoma, MIA）109例，浸润性腺癌（invasive adenocarcinoma, IAC）142例。对结节的影像形态学特征进行主观评价，并利用肺结节人工智能系统自动获得定量参数。**结果** 在主观CT影像学征象中，AAH+AIS、MIA和IAC组间结节最大径及毛刺征、分叶征、胸膜牵拉征出现的频率随病理级别增高而增加；在人工智能定量参数中，结节大小相关参数、CT值相关参数、实性占比、能量及熵随病理级别增高而增加。通过多因素Logistic逐步回归分析，人工智能定量参数在区分GGNs的病理类型中不亚于主观CT影像学征象。**结论** 人工智能定量参数对区分GGNs的病理类型有一定的价值。

## Abstract (en)

**Background and objective** The pathological types of lung ground glass nodules (GGNs) show great significance to the clinical treatment. This study was aimed to predict pathological types of GGNs based on computed tomography (CT) quantitative parameters. **Methods** 389 GGNs confirmed by postoperative pathology were selected, including 138 cases of precursor glandular lesions [atypical adenomatous hyperplasia (AAH) and adenocarcinoma in situ (AIS)], 109 cases of microinvasive adenocarcinoma (MIA) and 142 cases of invasive adenocarcinoma (IAC). The morphological characteristics of nodules were evaluated subjectively by radiologist, as well as artificial intelligence (AI). **Results** In the subjective CT signs, the maximum diameter of nodule and the frequency of spiculation, lobulation and pleural traction increased from AAH+AIS, MIA to IAC. In the AI quantitative parameters, parameters related to size and CT value, proportion of solid component, energy and entropy increased from AAH+AIS, MIA to IAC. There was no significant difference between AI quantitative parameters and the subjective CT signs for distinguishing the pathological types of GGNs. **Conclusion** AI quantitative parameters were valuable in distinguishing the pathological types of GGNs.

由于肺结节人工智能软件的普及，肺结节的检出率增加，尤其是磨玻璃结节（ground glass nodules, GGNs）， 早期腺癌往往以GGNs为主要表现形式^[1,2]^。虽然有研究表示存在磨玻璃成分的肺结节术后生存率优于实性结节^[3]^，但不同病理类型的预后也不尽相同，原位腺癌（adenocarcinoma in situ, AIS）和微浸润腺癌（microinvasive adenocarcinoma, MIA）患者的5年无病生存率接近100%，而浸润性腺癌（invasive adenocarcinoma, IAC）患者仅有40%-85%^[4]^。此外，肺腺癌的病理类型决定了手术方式，相较于非典型瘤样增生（atypical adenomatous hyperplasia, AAH）+AIS、MIA而言，目前IAC推荐在切除肺叶后行淋巴结采样或清扫^[5,6]^。另外，根据2021年世界卫生组织（World Health Organization, WHO）最新胸部肿瘤分类标准^[7]^，AAH、AIS已归为前驱腺体病变而非腺癌。因此，区分GGNs是否为AAH+AIS、MIA、IAC具有十分重要的临床意义。

由于人工智能软件在肺结节诊疗中的广泛使用，且具有高重复性的优点，有效地避免了人工测量误差以及主观观察不一致的问题，通过人工智能软件鉴别GGNs的病理类型具有临床实用价值。既往研究^[8⇓⇓⇓-12]^中不少学者利用人工智能参数有效地对AAH+AIS与MIA+IAC以及AAH+AIS+MIA与IAC进行了二分类，关于结节AAH+AIS、MIA、IAC的三分类相关研究较少，本研究旨在通过回顾性分析患者术前胸部计算机断层扫描（computed tomography, CT）图像，探讨人工智能定量参数在鉴别诊断GGNs病理类型中的价值。

## 1 资料与方法

### 1.1 研究对象

收集2019年1月至2023年5月南京医科大学附属苏州医院符合纳入标准的359例患者的389个GGNs，女性242例，男性117例，年龄19-80岁，平均年龄（52.33±12.49）岁，AAH+AIS 138例，MIA 109例，IAC 142例。纳入标准：（1）具有手术前2周以内胸部CT图像，图像质量良好，且具有1或1.25 mm薄层重建图像；（2）胸部CT图像上肺结节表现为GGNs，且结节最大径5-30 mm；（3）经过手术切除并经病理证实为AAH、AIS、MIA及IAC；（4）人工智能系统肺结节三维识别成功。

### 1.2 检查方法

采用荷兰飞利浦Brilliance iCT或Ingenuity螺旋CT进行常规胸部扫描，患者取仰卧位，双手抱头，吸气末屏气，由肺尖扫描至肺底，管电压120 kV，自动管电流，层厚5 mm，层间隔5 mm，重建层厚1或1.25 mm。

### 1.3 数据处理

由两位有丰富工作经验的放射科诊断医师共同评估主观CT影像学征象，人工测量横断面结节最大径，观察结节的位置及是否存在毛刺征、分叶征、空泡征、胸膜牵拉征、血管穿行征及支气管穿行征。将常规DICOM 格式CT图像导入推想医疗科技股份有限公司肺结节CT影像辅助检测软件（InferRead CT Lung 4.0），自动勾画结节三维边界，并进行结节分析（图1），由该系统自动计算出结节大小相关参数（包括体积、表面积、3D最大面面积、质量、3D长径）、CT值相关定量参数（包括CT最大值、CT最小值、平均CT值、CT中位数、CT值标准差）、实性占比、紧凑度、球形度、峰度、偏度、能量及熵。

**图1:**
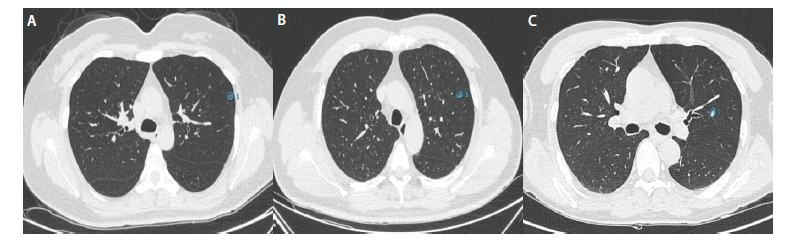
肺结节CT影像辅助检测软件自动识别肺结节并勾画边界。A：AIS；B：MIA；C：IAC。

### 1.4 统计学分析

应用SPSS 27.0软件进行统计学分析。符合正态分布的计量资料采用Mean±SD描述，组间比较采用独立样本t检验或单因素方差分析；不符合正态分布的计量资料采用中位数（P_25_, P_75_）描述，组间比较采用非参数检验；计数资料采用频数（百分比）描述，组间比较采用卡方检验分析。P<0.05为差异有统计学意义。使用受试者工作特征（reciever operating characteristic, ROC）曲线对计量资料进行统计学分析。采用多因素Logistic逐步回归分别建立主观CT影像学征象模型、人工智能参数模型以及两者结合的联合模型。

## 2 结果

### 2.1 AAH+AIS组、MIA组、IAC组主观CT影像学征象资料的比较

AAH+AIS组、MIA组、IAC组的结节最大径逐渐增大；毛刺征、分叶征、胸膜牵拉征在AAH+AIS组、MIA组、IAC组出现的频率依次增加；在结节位置上，AAH+AIS组、MIA组及IAC组三组差异无统计学意义（表1）。通过ROC曲线分析，AAH+AIS与MIA、MIA与IAC的结节最大径最佳临界值分别为7.50、12.50 mm。

**表1:** AAH+AIS组、MIA组、IAC组主观CT影像学征象资料比较

Index	AAH+AIS (n=138)	MIA (n=109)	IAC (n=142)	P
2D long diameter (mm)^abc^	7.00 (6.00, 9.25)	9.00 (7.00, 11.00)	10.00 (8.00, 16.00)	<0.001
Location				0.637
Right upper lobe	50 (36.23%)	35 (32.11%)	45 (31.69%)	
Right middle lobe	7 (5.07%)	3 (2.75%)	9 (6.34%)	
Right lower lobe	24 (17.39%)	20 (18.35%)	33 (23.24%)	
Left upper lobe	40 (29.99%)	39 (35.78%)	36 (25.35%)	
Left lower lobe	17 (12.32%)	12 (11.01%)	19 (13.38%)	
Spiculation^abc^				<0.001
Yes	4 (2.90%)	13 (11.93%)	41 (28.87%)	
No	134 (97.10%)	96 (88.07%)	101 (71.13%)	
Lobulation^abc^				<0.001
Yes	39 (28.26%)	45 (41.28%)	94 (66.20%)	
No	99 (71.74%)	64 (58.72%)	48 (33.80%)	
Vacuole^c^				0.039
Yes	24 (17.39%)	20 (18.35%)	41 (28.87%)	
No	114 (82.61%)	89 (81.65%)	101 (71.13%)	
Pleural traction^abc^				<0.001
Yes	23 (16.67%)	38 (34.86%)	73 (51.41%)	
No	115 (83.33%)	71 (65.14%)	69 (48.59%)	
Vascular passing-through sign^c^				0.046
Yes	130 (94.20%)	106 (97.25%)	141 (99.30%)	
No	8 (5.80%)	3 (2.75%)	1 (0.70%)	
Air bronchogram sign^ac^				0.003
Yes	6 (4.35%)	12 (11.01%)	24 (16.90%)	
No	132 (95.65%)	97 (88.99%)	118 (83.10%)	

^a^, ^b ^and ^c^ indicated that there were statistical differences (P<0.05) in AAH+AIS vs MIA, MIA vs IAC, and AAH+AIS vs IAC, respectively.

### 2.2 AAH+AIS组、MIA组、IAC组人工智能定量资料的比较

AAH+AIS组、MIA组及IAC组在结节大小相关参数（体积、表面积、3D最大面面积、质量、3D长径）、CT相关定量参数（CT最大值、CT最小值、平均CT值、CT中位数、CT值标准差）、实性占比、能量和熵中依次增大，在紧凑度、球形度中依次减小；在峰度、偏度中，AAH+AIS组、MIA组大于IAC组（表2）。通过ROC曲线分析，在区分AAH+AIS与MIA时，体积、表面积、3D最大面面积、质量、3D长径、CT最大值、CT最小值、平均CT值、CT中位数、CT值标准差、实性占比、紧凑度、球形度、峰度、偏度、能量及熵的曲线下面积（area under the curve, AUC）分别为0.64、0.64、0.63、0.69、0.65、0.64、0.63、0.65、0.64、0.62、0.61、0.58、0.58、0.55、0.56、0.59及0.65，质量分类效能最佳，峰度分类效能最差，平均CT值分类效能优于其他CT值相关定量参数；在区分MIA与IAC时，体积、表面积、3D最大面面积、质量、3D长径、CT最大值、CT最小值、平均CT值、CT中位数、CT值标准差、实性占比、紧凑度、球形度、峰度、偏度、能量及熵的AUC分别为0.63、0.64、0.64、0.66、0.65、0.63、0.66、0.69、0.67、0.69、0.68、0.67、0.66、0.74、0.71、0.57及0.69，峰度分类效能最佳，平均CT值、CT值标准差分类效能优于其他CT值相关定量参数（图2）。

**表2:** AAH+AIS组、MIA组、IAC组人工智能定量资料比较

Index	AAH+AIS	MIA	IAC	P
Volume (mm^3^)^abc^	181.00 (110.75, 362.19)	285.05 (159.63, 542.40)	527.29 (178.72, 1196.54)	<0.001
Surface area (mm^2^)^abc^	220.30 (168.13, 344.78)	302.00 (204.50, 462.25)	487.75 (235.10, 852.10)	<0.001
3D maximum area (mm^2^)^abc^	50.05 (38.20, 72.58)	63.10 (46.45, 96.00)	100.30 (53.15, 168.55)	<0.001
Weight (mg)^abc^	75.57 (46.75, 149.45)	124.83 (77.35, 237.04)	284.87 (98.17, 672.03)	<0.001
3D long diameter (mm)^abc^	10.25 (8.80, 12.53)	11.70 (9.95, 15.35)	15.50 (10.90, 21.33)	<0.001
Maximum CT value (HU)^abc^	-210.00 (-359.75, -10.00)	-48.00 (-245.50, 60.50)	45.50 (-134.25, 147.00)	<0.001
Minimum CT value (HU)^abc^	-788.50 (-821.00, -748.75)	-764.00 (-798.50, -720.50)	-724.50 (-772.75, -653.75)	<0.001
Mean CT value (HU)^abc^	-639.00 (-695.00, -576.25)	-578.00 (-653.00, -513.50)	-491.00 (-595.00, -401.50)	<0.001
Median of CT value (HU)^abc^	-663.00 (-712.5, -595.50)	-600.00 (-674.50, -541.50)	-540.50 (-622.50, -428.75)	<0.001
Standard deviation of CT value^abc^	106.80 (87.20, 134.06)	120.67 (99.74, 148.75)	152.37 (120.20, 186.07)	<0.001
Proportion of solid components (%)^abc^	0.00 (0.00, 0.80)	0.44 (0.00, 2.09)	2.78 (0.00, 11.92)	<0.001
Compactness (×10^-3^)^abc^	39.15 (35.68, 41.10)	37.60 (34.00, 40.50)	34.00 (31.15, 38.30)	<0.001
Sphericity^abc^	0.82 (0.77, 0.84)	0.79 (0.74, 0.84)	0.74 (0.70, 0.80)	<0.001
Kurtosis^bc^	-0.02 (-0.63, 1.40)	-0.19 (-0.63, 0.71)	-0.74 (-1.07, -0.32)	<0.001
Skewness^bc^	0.69 (0.44, 1.17)	0.63 (0.41, 0.84)	0.39 (0.14, 0.60)	<0.001
Energy (×10^8^)^abc^	2.12 (1.38, 3.93)	2.92 (1.69, 5.54)	3.90 (1.75, 8.95)	<0.001
Entropy^abc^	4.22 (3.95, 4.51)	4.44 (4.22, 4.73)	4.78 (4.44, 5.03)	<0.001

^a^, ^b ^and ^c^ indicated that there were statistical differences (P<0.05) in AAH+AIS vs MIA, MIA vs IAC, and AAH+AIS vs IAC, respectively.

**图2:**
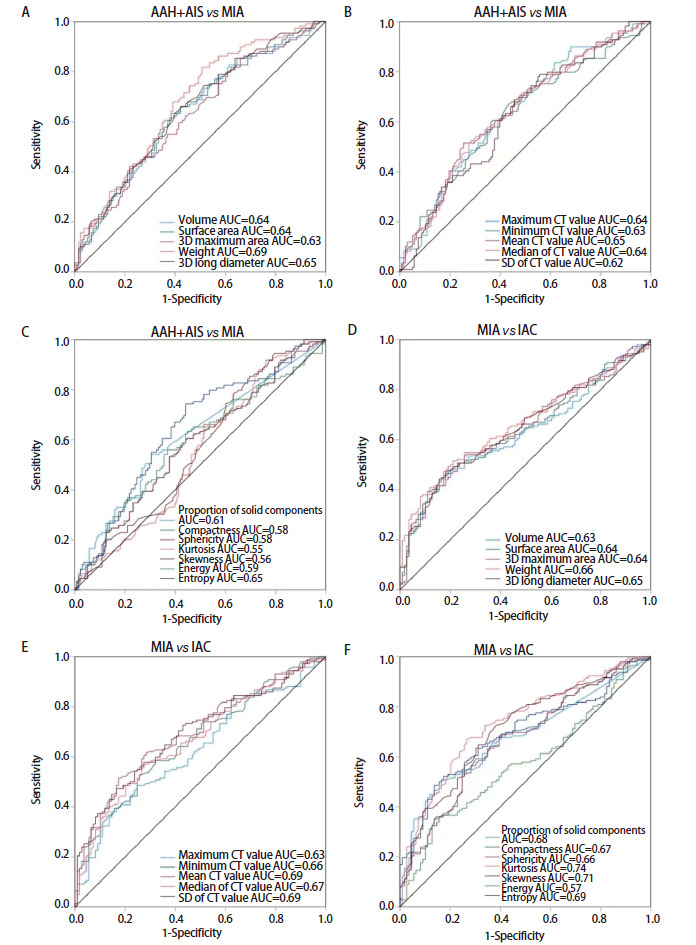
人工智能定量参数区分磨玻璃结节病理类型的ROC曲线。A-C：区分AAH+AIS与MIA的ROC曲线；D-F：区分MIA与IAC的ROC曲线。

### 2.3 Logistic逐步回归分析建立AAH+AIS组、MIA组、IAC组分类模型

AAH+AIS组、MIA组及IAC组三分类模型：主观CT影像学征象模型中，结节最大径、毛刺征、分叶征、胸膜牵拉征为参数，准确度为51.67%。人工智能定量参数模型中，3D长径、平均CT值、峰度为参数，准确度为56.56%。主观CT影像学征象、人工智能定量参数的联合模型中，3D长径、平均CT值、峰度为参数，而所有传统影像学参数均未进入模型，联合模型与人工智能定量参数模型为同一模型，准确度为56.56%（表3）。

**表3:** 主观CT影像学征象、人工智能定量参数及其联合预测AAH+AIS、MIA、IAC的多元Logistic回归模型结果

Model		Parameters	Coefficient	OR (95%CI)	Accuracy	Positive predictive value		
						AAH+AIS	MIA	IAC
Subjective CT signs	MIA	2D long diameter	-0.077	0.926 (0.862-0.994)	51.67%	74.64%	8.26%	62.68%
		Spiculation	0.553	1.739 (0.825-3.667)				
		Lobulation	0.559	1.748 (0.974-3.138)				
		Pleural traction	0.264	1.301 (0.741-2.287)				
	AAH+AIS	2D long diameter	-0.154	0.857 (0.786-0.935)				
		Spiculation	1.590	4.904 (1.589-15.128)				
		Lobulation	0.756	2.129 (1.182-3.834)				
		Pleural traction	1.016	2.762 (1.490 5.119)				
AI quantitative parameters	MIA	3D long diameter	-0.081	0.922 (0.872-0.975)	56.56%	78.99%	10.09%	70.42%
		Mean CT value	-0.004	0.996 (0.994-0.999)				
		Kurtosis	0.354	1.424 (1.023-1.983)				
	AAH+AIS	3D long diameter	-0.204	0.815 (0.758-0.877)				
		Mean CT value	-0.008	0.992 (0.989-0.995)				
		Kurtosis	0.408	1.504 (1.087-2.080)				
Combined	MIA	3D long diameter	-0.081	0.922 (0.872-0.975)	56.56%	78.99%	10.09%	70.42%
		Mean CT value	-0.004	0.996 (0.994-0.999)				
		Kurtosis	0.354	1.424 (1.023-1.983)				
	AAH+AIS	3D long diameter	-0.204	0.815 (0.758-0.877)				
		Mean CT value	-0.008	0.992 (0.989-0.995)				
		Kurtosis	0.408	1.504 (1.087-2.080)				

IAC was used as the reference. OR: odds ratio; CI: confidence interval.

AAH+AIS组、MIA+IAC组二分类模型：主观CT影像学征象模型中，结节最大径、毛刺征、胸膜牵拉征为参数，准确度为69.92%。人工智能定量参数模型中，3D长径、平均CT值为参数，准确度为73.52%。主观CT影像学征象、人工智能定量参数的联合模型中，胸膜牵拉征、3D长径、平均CT值为参数，准确度为72.75%（表4）。

**表4:** 主观CT影像学征象、人工智能定量参数及其联合参数预测AAH+AIS vs MIA+IAC、AAH+AIS+MIA vs IAC的二元Logistic回归模型结果

Index	Model	Parameters	Coefficient	OR (95%CI)	Accuracy
AAH+AIS vs MIA+IAC	Subjective CT signs	2D long diameter	0.145	1.156 (1.071-1.247)	69.92%
		Spiculation sign	-1.527	0.217 (0.074-0.641)	
		Pleural traction	-0.890	0.411 (0.237-0.710)	
	AI quantitative parameters	3D long diameter	0.166	1.181 (1.107-1.260)	73.52%
		Mean CT value	0.007	1.007 (1.005-1.010)	
	Combined	Pleural traction	-0.620	0.538 (0.302-0.957)	72.75%
		3D long diameter	0.150	1.162 (1.088-1.242)	
		Mean CT value	0.007	1.007 (1.004-1.009)	
AAH+AIS+MIA vs IAC	Subjective CT signs	2D long diameter	0.107	1.113 (1.044-1.186)	73.52%
		Spiculation	-0.893	0.409 (0.207-0.810)	
		Lobulation	-0.673	0.510 (0.308-0.846)	
		Pleural traction	-0.603	0.547 (0.332-0.901)	
	AI quantitative parameters	Surface area	0.002	1.002 (1.001-1.003)	75.84%
		Mean CT value	0.006	1.006 (1.004-1.008)	
		Kurtosis	-0.544	0.580 (0.373-0.902)	
		Skewness	0.523	1.688 (0.685-4.157)	
	Combined	Surface area	0.002	1.002 (1.001-1.003)	75.84%
		Mean CT value	0.006	1.006 (1.004-1.008)	
		Kurtosis	-0.544	0.580 (0.373-0.902)	
		Skewness	0.523	1.688 (0.685-4.157)	

AAH+AIS+MIA组、IAC组二分类模型：主观CT影像学征象模型中，结节最大径、毛刺征、分叶征、胸膜牵拉征为参数，准确度为73.52%。人工智能定量参数模型中，结节表面积、平均CT值、峰度、偏度为参数，准确度为75.84%。主观CT影像学征象、人工智能定量参数的联合模型与人工智能定量参数模型为同一模型，准确度为75.84%（表4）。

## 3 讨论

肺腺癌是早期肺癌最常见的病理类型，且以GGNs为主要表现形式。在2021年WHO最新胸部肿瘤分类标准中，AAH、AIS不再纳入肺癌分类，且非IAC与IAC手术方式存在差异，区分GGNs的病理类型对于临床诊疗中具有重要意义。近年来，人工智能发展迅速，在肺结节诊疗过程中发挥越来越大的作用，人工智能软件在识别肺结节的同时可以自动获取结节的定量资料，对术前预测肺结节的病理类型具有一定的价值。

肿瘤的最大直径是肿瘤病理侵袭性的重要危险因素，肿瘤的最大径与其侵袭性呈正相关。随着GGNs病理级别的增加，由于肿瘤组织成分增多，肺泡间隔增厚，病变累及的范围增大，结节的长径也随之增加。既往研究^[8,13,14]^中，GGNs的最大径对于AAH+AIS与MIA+IAC、非IAC与IAC二分类具有鉴别意义。本研究中GGNs最大径从AAH+AIS、MIA至IAC依次增大。Fang等^[15]^研究中AAH+AIS与MIA、MIA与IAC结节最大径最佳临界值为8.98、10.33 mm，而本研究中最佳临界值分别为7.50、12.50 mm，二者略有不同，这可能是人工测量的差异引起的；此外，与结节大小相关的定量参数（体积、表面积、3D最大面面积、质量、3D长径）在区分三分类中同样具有统计学意义，且从AAH+AIS、MIA至IAC逐渐增加。

CT值作为GGNs侵袭性的另一重要因素，由于肺结节的平均CT值与肿瘤细胞侵袭正常肺组织引起的肌成纤维细胞基质增厚程度相关，侵袭性越大，平均CT值越高。既往研究^[11,12]^中发现平均CT值能够有效预测肺腺癌的病理类型。本研究中CT值相关定量参数（最大值、最小值、平均值、中位数、标准差）随病理级别的增加而逐渐增大，此外，通过ROC曲线分析发现平均CT值对于分类AAH+AIS与MIA、MIA与IAC的AUC（0.65, 0.69）高于其他CT值相关定量参数，更能有效地鉴别GGNs的病理类型。

毛刺征、分叶征、空泡征、胸膜牵拉征、血管穿行征及支气管穿行征均为提示肺结节恶性的影像学征象，结节恶性程度越高，其出现的概率越大^[16,17]^。本研究进一步发现毛刺征、分叶征、胸膜牵拉征在AAH+AIS、MIA、IAC中出现的频率随病理级别的增高而逐渐增加，而空泡征、血管穿行征、支气管穿行征并没有该表现。既往研究^[9,10,18,19]^中也有发现空泡征、血管穿行征、支气管穿行征在鉴别GGNs病理类型中无统计学意义，空泡征、血管穿行征、支气管穿行征是否能够鉴别GGNs病理类型存在争议，仍需要进一步验证。

紧凑度与球形度衡量了结节形态，本研究中在AAH+AIS、MIA、IAC中逐渐减小，提示随着病理级别的增高，结节将不再近似于球体，形态越不规则。峰度、偏度分别代表结节中CT值的波动范围以及不对称性，即为反映结节密度均匀程度的参数。既往研究中^[12,20⇓⇓⇓-24]^峰度、偏度对GGNs病理类型具有预测价值。本研究中峰度、偏度在三组间存在差异，但在AAH+AIS、MIA组间不存在统计学差异，则表示AAH+AIS、MIA在CT值分布中存在较大相似性。熵衡量了图像纹理的复杂程度，病理级别越高，浸润成分越多，结节的纹理则表现得越复杂，熵值也就越大。Gao等^[23]^研究中熵是分类AIS+MIA、IAC的重要指标，本研究中熵从AAH+AIS、MIA至IAC递增。能量值衡量了图像中体素值的大小，本研究中发现病理级别越高，能量值越大，但在鉴别AAH+AIS与MIA、MIA与IAC的AUC（0.59, 0.57）较小，提示能量在预测GGNs病理类型方面可靠性可能不高，需要进一步加大样本量进行验证其诊断价值。

通过Logistic逐步回归分析发现，人工智能定量参数模型在GGNs三分类以及二分类的准确度上不亚于主观CT影像学征象模型，联合模型也并未明显提高分类的准确度；且在联合模型中，AAH+AIS、MIA、IAC三分类模型中并未纳入主观CT影像学征象，AAH+AIS、MIA+IAC二分类的联合模型中虽纳入了胸膜牵拉征参数，但并没有提高模型准确度，这表明人工智能定量参数对于GGNs病理类型的鉴别能力不亚于主观CT影像学征象，更有利于肺结节的临床自动化诊疗。此外，AAH+AIS、MIA、IAC三分类的主观CT影像学征象模型、人工智能定量参数模型及其联合模型中，MIA的阳性预测值极低，约90%的MIA被误认为是AAH+AIS及IAC，这可能是由于MIA是AAH+AIS发展为IAC的中间阶段，与AAH+AIS有一定的相似性，并存在进展为IAC的可能性。但本研究中多元Logistic逐步回归三分类模型相较于二分类模型的准确度不高，这可能需要通过影像组学、深度学习模型等进一步提取结节特征来进行分类。

本研究存在一定的局限性。首先，本研究属于回顾性研究，样本数据来源于单中心，样本可能存在偏倚；其次，三组样本量不均衡，临床实际中术后病理证实为AAH的GGNs样本量偏少。

综上所述，GGNs的人工智能定量参数在一定程度上能够区分GGNs的病理类型，能方便地为临床开展精准化以及个性化治疗提供参考依据。

**Competing interests**

The authors declare that they have no competing interests.

**Author contributions**

Shi YQ and Liu KF conceived and designed the study. Shi YQ, Shen YW and Yan WY collected patient data. Shi YQ, Shen YW and Chen J supported the statistical analysis. Shi YQ and Liu KF provided critical inputs on design, analysis, and interpretation of the study. All the authors had access to the data. All authors read and approved the final manuscript as submitted.
